# Modeling the Magnetoelectric Composites in a Wide Frequency Range

**DOI:** 10.3390/ma16175813

**Published:** 2023-08-24

**Authors:** Mirza Bichurin, Oleg Sokolov, Sergey Ivanov, Elena Ivasheva, Viktor Leontiev, Vyacheslav Lobekin, Gennady Semenov

**Affiliations:** Institute of Electronic and Information Systems, Yaroslav-the-Wise Novgorod State University, ul. B. St. Petersburgskaya, 41, 173003 Velikiy Novgorod, Russia; o-v-sokolov@mail.ru (O.S.); sivanovvad@mail.ru (S.I.); ellen9879@yandex.ru (E.I.); viktorsergeevich.novsu@gmail.com (V.L.); slavalobekin@gmail.com (V.L.); gennady.semenov@novsu.ru (G.S.)

**Keywords:** ferromagnetic metal, piezoelectric, magnetoelectric composite, magnetoelectric effect, magnetoelectric voltage coefficient, electro-mechanical resonance, resonance mode, ferromagnetic resonance line shift, substrate effect

## Abstract

This article presents a general theory of the ME effect in composites in the low- and high-frequency ranges. Besides the quasi-static region, the area of electromechanical resonance, including longitudinal, bending, longitudinal shear, and torsional modes, is considered in more detail. To demonstrate the theory, expressions of ME voltage coefficients are obtained for symmetric and asymmetric layered structures. A comparison is made with the experimental results for the GaAs/Metglas and LiNbO_3_/Metglas structures. The main microwave ME effect, consisting of the FMR line shift in an electric field, for the ferromagnetic metals, their alloys, and YIG ferrite using various piezoelectrics is discussed. In addition to analytical calculations, in the article, finite element modeling is considered. The calculation methods and experimental results are compared for some composites.

## 1. Introduction

Research of the magnetoelectric (ME) effect in the low-frequency and high-frequency ranges is given great attention in scientific periodicals, which is associated with the possibility of developing new electronic devices, such as current and magnetic field sensors, harvesters, microwave devices, etc. [1,2,3,4,5,6,7,8,9,10,11,12,13,14]. The main element of these new devices is a symmetric or asymmetric ME structure, from which it is required to obtain a sufficiently large ME effect, the magnitude of which is mainly determined by the parameters of its constituent components. Therefore, the development of each ME device begins with an analysis of the ME structure, on the basis of which it is planned to create a new device [15]. There are already many analytical and computer techniques that allow one to calculate the ME coefficients of various ME structures. This article attempts to collect and analyze basic information on various calculation methods.

A significant contribution to the development of the calculation of the ME effect in the low-frequency range was made by the analytical calculations of Harshe et al. [16]. Here, the ME coefficients for the direct ME effect were introduced based on the generalized Hooke’s law and their estimates were given. Another approach to the calculation of the ME effect was demonstrated by Nan [17], where he proposed to consider the ME behavior in the composite by a Green’s function method and perturbation theory and presented the theoretical estimates of ME coefficients for bulk composites. Subsequent works in this direction [18,19,20] were devoted to taking into account the directions of electric and magnetic fields, analyzing the effect of the interface on the magnitude of the ME coefficients, and reviewing the results obtained. The next important work was the work of Bichurin et al. [21], where they proposed to use the ME effect in the region of electromechanical resonance (EMR), which made it possible to increase the effect by 1–2 orders of magnitude. In Refs. [22,23,24,25,26,27,28,29], the authors presented calculations of the ME effect both at low frequencies and in various EMR modes: Longitudinal, bending, and shear. In addition to the analytical approach, Dong et al. [30,31] presented the results of calculating the ME coefficients by the equivalent circuit method. Later, analytical calculations of the ME coefficients were carried out for the inverse ME effect [32,33] and for the case of a gradient structure [34,35,36].

When studying ME composites in the low-frequency range, along with analytical methods, numerical methods of finite element modeling (FEM) are often used. Researchers use the FEM method both in conjunction with analytical methods and completely independently to fully calculate the ME effect. Some authors of such works use their own programs based on independently developed FEM algorithms. Others use specialized commercial software such as Ansoft 1.0 and Comsol Multiphysics 6.0. In Refs. [37,38], the authors use Ansoft, a commercial FEM magnetic field simulation program, to calculate the magnetic field in gradient magnetostrictive structures. In Refs. [39,40], the commercial software package FEM Comsol is used to calculate the ME effect. In Refs. [41,42,43,44,45,46], the authors use their own programs based on independently developed 2D FEM algorithms to calculate the ME effect. In Ref. [47], Comsol is used to calculate the magnetic field in the magnetostrictive phase of an ME composite. In Ref. [48], the authors use Comsol to calculate the magnetic field, strain distribution, and resonant frequency. In Refs. [49,50], Comsol is used for the FEM simulation. In Ref. [51], the authors use Comsol for FEM of the static ME voltage coefficient in magnetostrictive–piezoelectric structures with 1–3 and 0–3 connectivity.

In the high-frequency region, the attention of researchers was attracted by the ME effect in the layered YIG/PZT structure, which manifests itself as a shift of the FMR line in the magnetostrictive phase when a constant electric field is applied to the piezoelectric phase [52]. Subsequently, this type of ME effect was called the microwave ME effect. A consistent theory of the microwave ME effect was developed by Bichurin et al. [53]. They introduced the concept of the ME constant for the microwave ME effect and estimated its value on the basis of experiments [54]. Subsequently, several groups of authors proposed the use of the microwave ME effect in new microwave devices such as filters, phase shifters, valves, etc. [55,56,57]. H.-M. Zhou and J. Lian in [58] applied the equivalent circuit method in designing tunable ME microwave devices. Relatively recently, an interesting feature of the dependence of the FMR line shift under the action of an electric field on the direction of the bias field relative to the crystallographic axes of the magnetostrictive phase was discovered. As it turned out, the magnitude of the shift of the FMR line for some crystallographic orientations of the bias field exceeds the effect obtained when the bias field is directed along the main crystallographic axes [59,60,61,62,63]. To explain this feature, T. Nan et al. [59] proposed to take into account the surface charge for the Permalloy/PMN-PT structure, and W. Hou et al. [61] explained a similar result in the MnZn/PMN-PT structure by the two-magnon scattering process. The authors of [64,65,66,67] studied theoretically and experimentally the microwave ME effect in ferrite–piezoelectric and ferromagnetic metal–piezoelectric structures. A rather large experimental value of the microwave ME effect on the structures of Fe_3_O_4_ and FeGaB with PZN-PT has been obtained, which makes it possible to approach practical application in microwave ME devices. In Ref. [67], the authors applied analytical methods for the theoretical study of ME structures consisting of various ferromagnetic metals (Ni, Fe, Co, NiFe, FeGaB), as well as YIG ferrite and piezoelectrics (quartz, PZT, PMN-PT, PZN-PT). The effect of the possible presence of a GGG substrate has been theoretically studied in sufficient detail. When studying ME composites in the microwave range, along with analytical methods, numerical estimations of FEM were also used. The researchers used commercial software products such as High-Frequency System Simulator (HFSS) Ansys 15.0, Comsol Multiphysics 6.0, and CST Studio 2022. In Ref. [68], the authors used HFSS for electromagnetic FEM simulation of the microwave ME effect. In Ref. [69], Comsol and CST Studio, were used to simulate the characteristics of an ME antenna in the microwave range. In Ref. [70], the authors conducted a study for the design of elements of the ME microwave isolator using HFSS program for electromagnetic FEM simulation. In Ref. [71], the authors used Comsol to study the microwave ME effect.

## 2. Low-Frequency Range

### 2.1. Analytical Methods

This section discusses previous studies of the direct and inverse magnetoelectric effects in multilayer piezoelectric/magnetostrictive material composites at low frequencies and at the electromechanical resonance (EMR) frequency. In this section, in the studies under consideration for theoretical calculations and measurements of the ME voltage coefficient, two-layer and three-layer composites in the form of thin rectangular plates are used. Magnetostrictive materials used in the studies under consideration for theoretical calculations are cobalt ferrite, nickel ferrite or lanthanum-strontium manganite, Metglas, Terfenol-D, YIG, and as piezoelectrics are used barium titanate, PZT, gallium arsenide, and lithium niobate. Summarizing the results of the studies performed on the ME effect, we can draw the following conclusions: At a certain ratio of the volumes of the piezoelectric and magnetic materials and a certain value of the constant magnetizing field, the ME voltage coefficient takes the maximum value; at the EMR frequency of the composite, the voltage coefficient is much greater than at low frequencies; with mechanical fixation of one end of the composite and the free second end of the composite, the resonant frequency of the ME effect is much lower than for a free composite.

G. Harshe et al. in Ref. [16] considered the low-frequency direct ME effect in two-layer ME composites, while the theoretical calculation of the ME voltage coefficient assumes ideal coupling at the interface of the composite phases. The main disadvantages of this theoretical calculation are that the effects associated with the finite magnetic permeability of the ferrite are not taken into account; the transverse orientation of the electric and magnetic fields is not considered, at which a large value of the ME voltage coefficient is observed, and the non-ideal connection of the contacting surfaces of the phases of the composites is not taken into account. Another approach to the calculation of the ME effect was demonstrated by Nan [17], where he proposed to consider the ME behavior in the composite by a Green’s function method and perturbation theory and presented the theoretical estimates of ME coefficients for bulk composites. Later, in Refs. [18,19,20], a theoretical model of the low-frequency direct magnetoelectric effect in two-layer magnetostrictive material/piezoelectric composites was presented. In these theoretical calculations, the non-ideal mechanical phase coupling was taken into account by introducing the phase coupling coefficient into the calculation of the ME voltage coefficient; with a decrease in the phase coupling coefficient, the ME voltage coefficient also decreased. The calculation was performed for free and mechanically fixed composites at one end and three different orientations of magnetic and electric fields: Longitudinal orientation, transverse orientation, and longitudinal orientation in the plane of the composite. The theoretical calculation of the longitudinal and bending ME effects at the EMR frequency of layered ME composites depending on the frequency of an alternating magnetic field was given in the works [21,22,23]. In Ref. [1], in addition to composites in the form of a rectangular thin plate, the direct ME effect in the EMR region is considered for composites having the shape of a thin disk. A theoretical model for calculating the ME voltage coefficient at low frequencies is presented by D. Hasanyan et al. in Refs. [24,25,26,27,28], depending on the volume ratio of the magnetic phase to the volume of the ME composite. In Ref. [28], the theoretical calculation was carried out taking into account the influence of electrodes and several epoxy layers; the results of measurements of the ME coefficient by voltage were in good agreement with the theoretical calculation. It was determined that with a thinner Kapton film with electrodes and thin epoxy layers connecting the phases of the composite, the value of the ME voltage coefficient increased. In Ref. [24], the optimal number of Metglas films was determined, at which the ME voltage coefficient for the considered composite is at its maximum. The dependence of the ME voltage coefficient on the magnitude of the permanent magnetic field during bending and longitudinal vibrations of the ME composite at low frequencies was considered in [26]. It was determined that at a constant magnetic field, at which saturation of the magnetostriction of the magnetic material occurs, the ME voltage coefficient is at its maximum. To study the dependence of the ME voltage coefficient for the longitudinal and bending modes of the ME effect at low frequencies on the geometric dimensions of the ME composite phases, a calculation method was developed by Y. Wang et al. in [25]. In the developed model, the ratios of the length, width, and thickness of the piezoelectric layer to the magnetostrictive layer were used to predict the change in the ME voltage coefficient, which depends on the geometry of the composite. Predictions show that, in addition to the material parameters of the composite phases, the geometric parameters of length, width, and phase thickness ratio also significantly affect the ME voltage coefficient. An analytical model that also takes into account the effect of shear lag in the calculation of the direct ME effect at the EMR frequency was developed in [26,27]. The results of the calculations show that the shear lag causes a significant deformation inhomogeneity near the free ends of the ME composite, which reduces the value of the ME stress coefficient. The article [29] investigates the ME effect in the shear mode of oscillations along the thickness in a magnetostrictive–piezoelectric composite. The optimal geometric dimensions of the phases of the composite to increase the ME voltage coefficient are studied, while the theoretical calculation for the langatate Y-cut/YIG composite satisfactorily agrees with the measurements. In Refs. [30,31], S. Dong et al. determined the effective voltage gain and the output efficiency of ME composites. Research results show that ME composites provide a high output voltage gain at the EMR frequency relative to the applied voltage to the coil used to create an alternating magnetic field. The authors suggest that with a high voltage gain for ME composites, it is possible to use these composites in power transformers. It has also been determined that the maximum efficiency of magnetic field energy conversion by the ME composite is ~98%. In addition to the direct ME effect, the inverse ME effect is also studied. The inverse ME effect is theoretically and experimentally considered in the articles [1,32,33] at low frequencies and at the EMR frequency. The optimal constant biasing field and the ratio of the volumes of the piezoelectric to the volume of the composite, at which the maximum value of the inverse ME coefficient is observed, are determined. In Refs. [34,35], the use of an ME composite with a magnetostrictive phase made of a material with a saturation magnetization gradient and a ferroelectric phase made of a material with a polarization gradient is studied in various devices: Magnetic field sensors, tunable microwave filters, microwave delay lines, etc. A calculation is presented showing that the magnetostrictive phase of a material with a saturation magnetization gradient makes it possible not to use an external permanent magnetic bias field applied to the magnetostrictive phase of the ME composite to implement the ME effect. A theoretical calculation, taking into account the results of previous studies of the direct ME effect, is given in [15]. In addition to longitudinal and bending vibrations, longitudinal-shear and torsional vibrations were also considered for symmetric and asymmetric ME composites in the region of EMR and low frequencies. Based on the results of theoretical calculations in [15], the following conclusions were made: For the torsional mode of the ME effect during rotation of a two-layer ME composite along the length the ME voltage coefficient is an order of magnitude greater than during rotation along the width of the composite; the ME voltage coefficient at low frequencies in the longitudinal-shear vibration mode is less than in the longitudinal vibration mode and has the same form of dependence on the piezoelectric volume fraction as for the longitudinal vibration mode; it has been determined that the accuracy of the coincidence of the resonant frequencies with the theoretical values of the EMR frequencies depends on the material parameters of the composite phases for the vibration modes under consideration.

Below is a brief analytical calculation of the ME voltage coefficient for various vibration modes of the composite at low frequencies and in the EMR region according to [15]. We will consider the ME composite in the form of a thin, narrow plate, in which the thickness and width are much less than the length. Under these conditions, for the longitudinal and longitudinal-shear modes, the one-dimensional equations used below, which take into account only the measurement of length, are sufficiently accurate. For the bending mode, the two-dimensional equations used below, which take into account measurements of the length and thickness, are quite adequate. If the length and width of the ME composite are comparable, then the equations should additionally take into account the measurement of the width and be two-dimensional and three-dimensional, respectively. This case has not yet been studied theoretically and is not widespread in practice. The torsional mode calculation then uses fully 3D equations to account for length, width, and thickness measurements. For symmetrical ME composites, magnetostrictive layers of the same thickness are connected to the upper and lower faces of the piezoelectric layer; for asymmetric composites, magnetostrictive layers are located above the piezoelectric layer. The magnetic fields are directed along the length of the composite for longitudinal and bending modes and along the width of the composite for longitudinal-shear and torsional modes.

**Figure 1:**
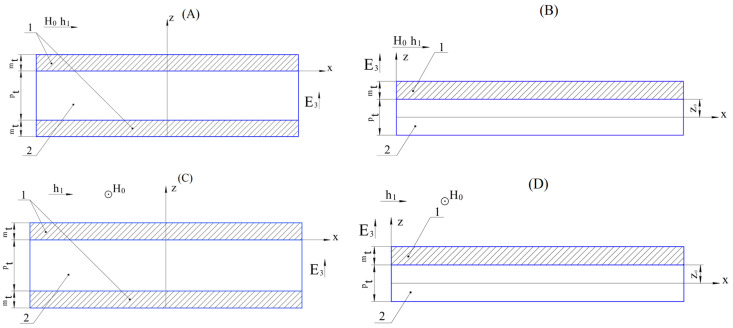
The structures of composites for analytical calculate of the ME voltage coefficient for various vibration modes of the composite: longitudinal mode (**A**), bending mode (**B**), longitudinal-shear mode (**C**) and torsional mode (**D**).

In Figure 1, 1 is a magnetostrictive phase, and 2 is a piezoelectric phase. ^p^t and ^m^t are the thicknesses of the piezoelectric and magnetostrictive phases; z_0_ is the distance between the lower face of the magnetostrictive phase and the neutral line of the asymmetric ME composite (B); z_0_ is the distance between the lower face of the magnetostrictive phase and the axis of rotation of the asymmetric ME composite (D); h_1_ is the alternating magnetic field; H_0_ is the permanent magnetic field; and E_3_ is the electric field in a piezoelectric.

#### 2.1.1. Longitudinal Mode

The transverse component of the electric displacement vector and the longitudinal component of the mechanical stress of the piezoelectric phase:

(1) D3=εε0E3+d31Tp1,

(2) Tp1=1sp11Sp1−d31sp11E3,

For the magnetostrictive phase, the longitudinal component of the stress tensor has the form:

(3) Tm1=YmBSm1−q¯11h1,

where *h*_1_ is the external alternating magnetic field away from the ME composite:

(4) q¯11=YmBq11,

Young’s modulus of the magnetostrictive phase at permanent magnetic induction:

(5) YmB=Ym1−K112m,

The square of the magnetomechanical coupling factor has the form:

(6) Km112=Ymq112μμ0,

Further, to determine the expression for calculating the ME voltage coefficient, an ideal mechanical contact between the magnetostrictive and piezoelectric phases is assumed, which causes an ideal transfer of mechanical deformations from the magnetostrictive phase to the piezoelectric one. In practice, the magnetostrictive and piezoelectric phases are connected with glue or some other method. In addition, the mechanical contact between the magnetostrictive and piezoelectric phases under experimental conditions is of different quality. In this regard, the experimental value of the ME effect is always less than the theoretical value, sometimes significantly, if the quality of the mechanical connection differs from the ideal one.

The longitudinal component of the stress tensor of the ME composite is:

(7) T1=νmTm1+νpTp1=c11S1−νmq¯11h1−νpd31sp11E3,

where volume fractions of the piezoelectric and magnetostrictive phases for a symmetric ME composite:

(8) νp=tptp+2tmνm=2tmtp+2tm,

For asymmetric ME composites, factor 2 should be removed before ^m^*t* in Equation (8) for the effective stiffness of the composite:

(9) c11=νpsp11+νmYmB,

effective density of the composite:

(10) ρ=νpρp+νmρm,

The solution of the motion equation for longitudinal deformations $\rho \frac{\partial^{2}U_{x}}{\partial \tau^{2}}=\frac{\partial T_{1}}{\partial x}$ is obtained as:

(11) $$U_{x}=A\cos\left(kx\right)+B\sin\left(kx\right),$$

where *A* and *B* are unknown constants, and wave number for longitudinal mode:

(12) $$k=\sqrt{\frac{\rho }{c_{11}}}\omega ,$$

The boundary conditions for free fixing of both ends of the composite:

(13) $$\begin{array}{l} {\left.T_{1}\right|}_{x=-\frac{l}{2}}=0\\ {\left.T_{1}\right|}_{x=\frac{l}{2}}=0 \end{array},$$

Solving the system of Equation (13), we find the values of the constants *A* and *B*. Next, we define the transverse component of the electric field strength vector through the following expression for an open circuit:

(14) $$\int_{-\frac{l}{2}}^{\frac{l}{2}}D_{3}dx=0,$$

Expressing *E*_3_ from Equation (14) after integrating, we find the ME voltage coefficient, taking into account the fact that the electric field exists only in the piezoelectric phase:

(15) αE=E3νph1=−νmνpq¯11d31sp11tanηεε0sp112c11η+d312νptanη−c11sp11η,

where $\eta =\frac{kl}{2}$.

#### 2.1.2. Bending Mode

The bending mode arises in asymmetric ME composites. The full thickness of the asymmetric Bending mode arises in asymmetric ME composites. The full thickness of the asymmetric composite:

(16) t=tp+tm,

Volume fractions of the piezoelectric and magnetostrictive phases for the asymmetric ME composite, respectively:

(17) νp=tptνm=tmt,

For the piezoelectric and magnetostrictive phases of the ME composite, the longitudinal components of the stress tensor and the transverse component of the electric field strength of the piezoelectric phase:

(18) Tp1=c11DS1−h31D3,

(19) Tm1=−zYmB∂2w∂x2−q¯11h1,

(20) $$E_{3}=-h_{31}S_{1}+\beta_{33}^{S}D_{3},$$

where:

(21) c11D=sp11E−d312ε33ε0−1h31=c11Dd31ε33ε0β33S=1+h31d31ε33ε0,

Bending moment:

(22) M=∫z0−tpz0bzTp1dz+∫z0z0+tmbzTm1dz=−b∂2w∂x2D−btp2h31D3−btm2q11h1,

where *b* is composite width:

(23) h31=1tp2∫z0−tpz0zh31dz=2z0−tp2tph31q11=1tm2∫z0z0+tmzq¯11dz=q¯112z0+tm2tm,

Full cylindrical stiffness of the composite:

(24) D=13c11Dtptp2−3tpz0+3z02+YmBtmtm2+3tmz0+3z02,

Electrical voltage in a piezoelectric phase of the composite:

(25) U=∫z0−tpz0E3dz=tp2h31∂2w∂x2+tpβ33SD3,

By expressing from Equation (25) the electrical displacement in the piezoelectric *D*_3_ and substituting it into Equation (22), we obtain:

(26) M=−bt3c11∂2w∂x2−btph31β33SU−btm2q11h1,

where:

(27) c11=1t3D−tp3h312β33S,

The distance between the lower face of the magnetostrictive phase and the neutral line of the asymmetric ME composite *z*_0_ is determined from the condition $\partial \langle c_{11}\rangle /\partial z_{0}=0$:

(28) z0=c11Dpt2−YmBmt2β33S−h312pt22YmBmt+c11Dptβ33S−h312pt,

The general solution of the flexural vibration equation $\rho bt\frac{\partial w^{2}}{\partial \tau^{2}}=\frac{\partial^{2}C}{\partial x^{2}}$ is obtained as:

(29) $$w=C_{1}\cosh\left(kx\right)+C_{2}\sinh\left(kx\right)+C_{3}\cos\left(kx\right)+C_{4}\sin\left(kx\right),$$

where *C*_1_, *C*_2_, *C*_3_, and *C*_4_ are unknown constants. The wave number for bending mode:

(30) $$k={\left(\frac{\rho }{t^{2}\langle c_{11}\rangle }\omega^{2}\right)}^{\frac{1}{4}},$$

Integrating Equation (25) over *x* considering the open circuit condition $\int_{0}^{l}D_{3}dx=0$, we obtain:

(31) Ul=tp2h31∂w∂x0l=tp2h31kC1r2+C2r1−1−C3r4+C4r3−1,

where:

(32) $$r_{1}=\cosh\left(kl\right),\;\;\;r_{2}=\sinh\left(kl\right),\;\;r_{3}=\cos\left(kl\right),\;\;r_{4}=\sin\left(kl\right),$$

For ME composites with free ends, the boundary conditions have the form:

(33) $$\begin{array}{l} \frac{\partial M}{\partial x}\left(0\right)=0,\;\;\frac{\partial M}{\partial x}\left(l\right)=0\\ M\left(0\right)=0,\;M\left(l\right)=0 \end{array},$$

Solving the system of Equations (31) and (33), we can find the values of the constants *C*_1_–*C*_4_ and voltage *U* in the piezoelectric phase of the composite. Then the ME voltage coefficient:

(34) αE=Uh1t=2tm2tp2q11h31β33Sr1r4+r2r3−r2−r4tc11klt3β33S1−r1r3−2tp3h312r1r4+r2r3−r2−r4,

In practice, in order to excite the bending mode of the ME effect, cantilever fastening of the ME composite is often used, when one end is rigidly clamped and the other is free. The calculation for this case differs only in the boundary conditions for the pinched end, and the final expression for the ME voltage coefficient has the form:

(35) αE=−tm2tp2q11h31β33Sr1r4+r2r3c11klt3β33S1+r1r3+tp3h312r1r4+r2r3t,

#### 2.1.3. Longitudinal-Shear Mode

For the longitudinal-shear mode, the equations for calculating the ME stress coefficient are the same as for the longitudinal mode, but the mechanical stress tensors are replaced by ^m^*T*_1_, ^p^*T*_1_ on ^m^*T*_6_, ^p^*T*_6_, piezoelectric and piezomagnetic modulus *d*_31_, *q*_11_ on *d*_36_, *q*_16_, compliance factor of the piezoelectric phase ^p^*s*_11_ on ^p^*s*_66_, Young’s modulus of magnetostrictive phases ^m^*Y*^B^ on shift modulus ^m^*G*, and effective coefficient of composite stiffness *c*_11_ on *c*_66_. For asymmetric ME, composite factor 2 should be removed before ^m^*t* in Equation (8).

The component of the strain tensor for longitudinal-shear vibrations, considering the ideal mechanical connection between the phases:

(36) Sm6=Sp6=S6=∂Uy∂x,

Then the ME voltage coefficient, considering the fact that the electric field exists only in the piezoelectric phase for free-fixing ME composites, will be equal to:

(37) αE=E3νph1=−νmνpq¯16d36sp66tanηεε0sp662c66η+d362νptanη−c66sp66η,

#### 2.1.4. Torsional Mode

A torsional mode arises in asymmetric ME composites. The full thickness t of the asymmetric ME composite is determined from Equation (16).

Shear components of the strain tensor for composites are:

(38) $$\begin{array}{l} S_{5}=y\frac{\partial \theta }{\partial x}\\ S_{6}=-z\frac{\partial \theta }{\partial x} \end{array},$$

where θ is the twist angle.

Tangent components of the piezoelectric phase stress tensor and electrical displacement are:

(39) pT5=GpS5=Gpy∂θ∂x,

(40) pT6=−GpDz∂θ∂y−h36D3,

(41) D3=−d36pGz∂θ∂x+εε0−Gpd362E3,

where:

(42) GpD=εε0Gpεε0−Gpd362h36=d36Gpεε0−Gpd362,

Tangent components of the magnetostrictive phases stress tensor are:

(43) mT5=GmS5=Gmy∂θ∂xmT6=GmS6−q16h1=−Gmz∂θ∂x−q¯16h1,

where:

(44) q¯16=Gmq16,

The torque is:

(45) M=∫−b2b2dy∫z0−tpz0yTp5−zTp6dz+∫−b2b2dy∫z0z0+tmyTm5−zTm6dz==K∂θ∂x+btp2h36D3+btm2q16h1,

where:

(46) K=Kp+KmKp=13GpDz03−z0−tp3b+112Gptpb3Km=Gm13z0+tm3−z03b+112tmb3,

$\langle h_{36}\rangle ,\;\langle q_{16}\rangle$ are determined from Equation (23), but *h*_31_, *q*_11_ are replaced by *h*_36_, *q*_16_. By expressing from Equation (41) *E*_3_, find the electrical voltage on the piezoelectric phase:

(47) U=∫z0−tpz0E3dz=tp2h36∂θ∂x+tpβ33SD3,

where:

(48) β33S=1εε0−Gpd362,

By expressing from Equation (47) the electrical displacement *D*_3_ in piezoelectric and substituting in Equation (45), we get:

(49) M=−bt3G∂θ∂x−btph36β33SU+btm2q16h1,

where:

(50) G=1bt3K−btp3h362β33S,

The position of the interface between the piezoelectric and magnetostrictive phases relative to the axis of rotation of the composite beam *z*_0_ is determined from the condition of the minimum effective shear modulus of the sample $\langle G\rangle$ according to Equation (28), but h_31_ is replaced by *h*_36_, *c*_11_^D^ is replaced by ^p^*G*^D^, ^m^*Y*^B^ is replaced by ^m^*G*, and β_33_^S^ is determined from Equation (48).

The solution of the motion equation for torsional vibrations $J\frac{\partial \theta^{2}}{\partial \tau^{2}}=\frac{\partial M}{\partial x}$ is obtained as:

(51) $$\theta =A\cos\left(kx\right)+B\sin\left(kx\right),$$

where *A* and *B* are unknown constants.

The wave number for torsional mode:

(52) $$k=\omega \sqrt{\frac{J}{bt^{3}\langle G\rangle }},$$

where the moment of inertia of the sample per unit width has the form:

(53) J=ρpIp+ρmIm,

The polar moments of the piezoelectric ^p^*I* and magnetostrictive phases ^m^*I* are determined from Equation (46) such as ^p^*K* and ^m^*K*, respectively, but ^p^*G*^D^, ^p^*G*, and ^m^*G* should be removed in Equation (46).

Boundary conditions for a free sample are:

(54) $$M\left(\frac{l}{2}\right)=0,\;M\left(-\frac{l}{2}\right)=0,$$

Then, we combine Equations (14), (47), and (54) into a system of equations.

Solving this system, we find the voltage on the piezoelectric *U* and then the ME voltage coefficient:

(55) αE=Uth1=2tp2tm2h36q16β33Stanηt2klt3Gβ33S+2h362tp3tanη,

To take into account energy losses during EMR, we set $\omega =2\pi \left(1+\frac{1}{Q}i\right)f$, where Q is the quality factor of the resonance. The value of the quality factor for longitudinal and bending modes, *Q* = 130. The dimensions of the ME composite are as follows: *l* = 1 × 10^−2^ m, *b* = 3 × 10^−4^ m, tp=5×10−4 m, tm=29×10−6 m. The value of the quality factor of the EMR for longitudinal-shear mode and torsional mode is *Q* = 300. Material of the piezoelectric layer: PZT, LN cut y + 128° for longitudinal and bending modes, and GaAs for longitudinal-shear and torsional modes. The material of the magnetostriction layer is Metglas.

**Figure 2:**
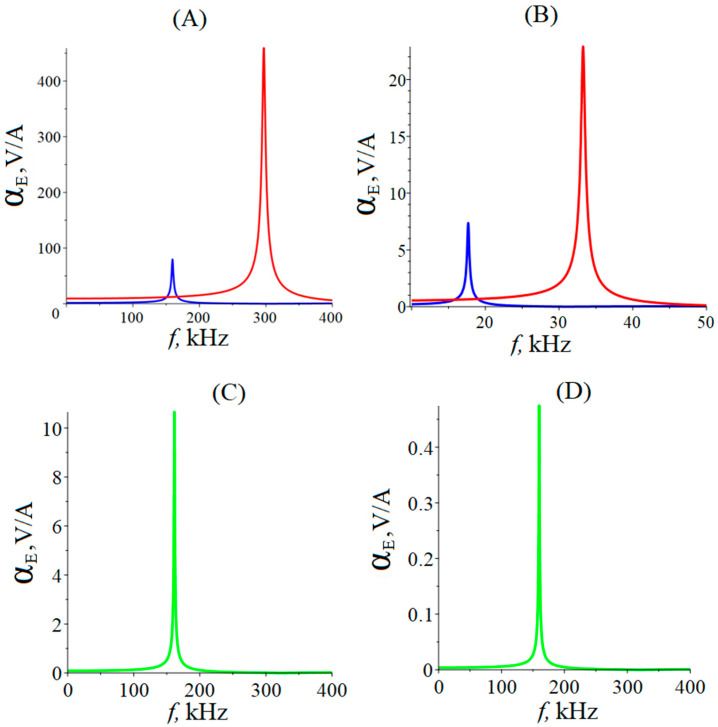
Dependence of the ME voltage coefficient on the frequency of an alternating magnetic field applied to an ME composite for longitudinal mode (**A**), bending mode (**B**), longitudinal-shear mode (**C**), torsional mode (**D**). Blue line is for PZT layer, red line is for LN cut y + 128° layer, green line for GaAs layer.

It can be seen from Figure 2 that the maximum value of the ME voltage coefficient for equal composite sizes for LN cut y + 128° is greater than for PZT since it has a larger ratio of the piezoelectric modulus to the relative permittivity of the piezoelectric material. For the bending mode, the maximum value of the ME voltage coefficient for LN cut y + 128° is greater than for PZT, for the same reason as for the longitudinal mode. The resonant frequency of the ME voltage coefficient for the longitudinal mode is nine times greater than for the bending mode. Moreover, the main resonant frequency of the bending mode with cantilevered fixing is much lower than with a free one. In this case, the maximum values of the ME voltage coefficient are only slightly less than in the case of free composite ends. Resonant frequencies for longitudinal-shear and torsional modes are equal, but the magnitude of the ME voltage coefficient for the longitudinal-shear mode is twenty times greater than for the torsional mode.

#### 2.1.5. Quasi-Static Regime

The magnitude of the ME coefficients for the quasi-static regime is determined by the values of the piezoelectric and piezomagnetic coefficients for the individual phases of the composite, the values of the compliance and stiffness coefficients of each phase, as well as the efficiency of the transfer of mechanical stresses between the individual phases of the composite. As mentioned earlier, the value of the ME voltage coefficient at low frequencies is lower than in the EMR region.

Below is an analytical calculation of the ME voltage coefficient for the low-frequency ME effect for various vibration modes of the composite in Figure 1.

#### 2.1.6. Quasi-Static Regime of Longitudinal and Longitudinal-Shear Modes for Symmetric ME Composites

Assuming in Equations (15) and (37) that the frequency f is equal to zero, we obtain the equations for the ME voltage coefficient in the quasi-static regime of the longitudinal and longitudinal-shear modes for symmetric composites:

(56) αE=νmνpq¯11d31νmYmBd312−εε0sp11c11,

(57) αE=νmνpq¯16d36νmGmd362−εε0sp66c66,

#### 2.1.7. Quasi-Static Regime for Asymmetric ME Composites

In an asymmetric composite, in the quasi-static regime of the ME effect and the orientation of a constant magnetic field directed along the length of the composite, both longitudinal and bending vibration modes arise simultaneously.

For the quasi-static regime, the motion equation of the bending vibrations of the ME composite has the form:

(58) $$\frac{\partial T_{1}}{\partial x}=0,$$

For the quasi-static regime, both longitudinal and bending modes are excited in the asymmetric ME structure, and the longitudinal strain tensor does not depend on x and takes the form:

(59) $$S_{1}=A+zB,$$

The first condition for the static equilibrium of an ME composite is that the total longitudinal force is equal to zero:

(60) ∫z0−tpz0Tp1dz+∫z0z0+tmTm1dz=0,

The second condition for the static equilibrium of the ME composite is the zero total moment, which is given by:

(61) ∫z0−tpz0zTp1dz+∫z0z0+tmzTm1dz=0,

Then we combine Equations (60) and (61) into a system of equations. Solving this system, we obtain *A* and *B*.

Substituting Equation (59) in Equation (20) and taking into account that due to the open circuit condition in Equation (14), we get:

(62) $$E_{3}=-h_{31}\left(A+zB\right),$$

Then, the voltage across the piezoelectric is:

(63) U=∫z0−tpz0−h31Aq+zBqdz=−h31Aqtp+12Bqtp2z0−tp,

Substituting the values found from the system of equations *A* and *B* in Equation (63), find the voltage on the piezoelectric and get the final expression for the ME voltage coefficient:

(64) αE=−tmtpc11Dtp3+YmBtm3⋅c11D2tp4+4c11DYmBtptm3+6c11DYmBtp2tm2+⋅h31q¯11+4c11DYmBtmtp3+YmB2tm4tm+tp,

In an asymmetric composite in the quasi-static regime of the ME effect, with the orientation of a constant magnetic field *H*_0_ along the width of the composite, longitudinal-shear and torsional modes arise simultaneously. The strain tensors must not depend on x, and the strain tensors have the form.

(65) $$\begin{array}{l} S_{5}=yB\\ S_{6}=A-zB \end{array},$$

The first condition for the static equilibrium of an ME composite with the orientation of a constant magnetic field *H*_0_ along the width of the composite has the form:

(66) ∫z0−tpz0Tp6dz+∫z0z0+tmTm6dz=0,

The second condition for the static equilibrium of the ME composite is zero torque, given by:

(67) ∫−b2b2dy∫z0−tpz0yTp5−zTp6dz+∫−b2b2dy∫z0z0+tmyTm5−zTm6dz=0,

Then we combine Equations (66) and (67) into a system of equations. Solving this system, we obtain *A* and *B*.

**Figure 3:**
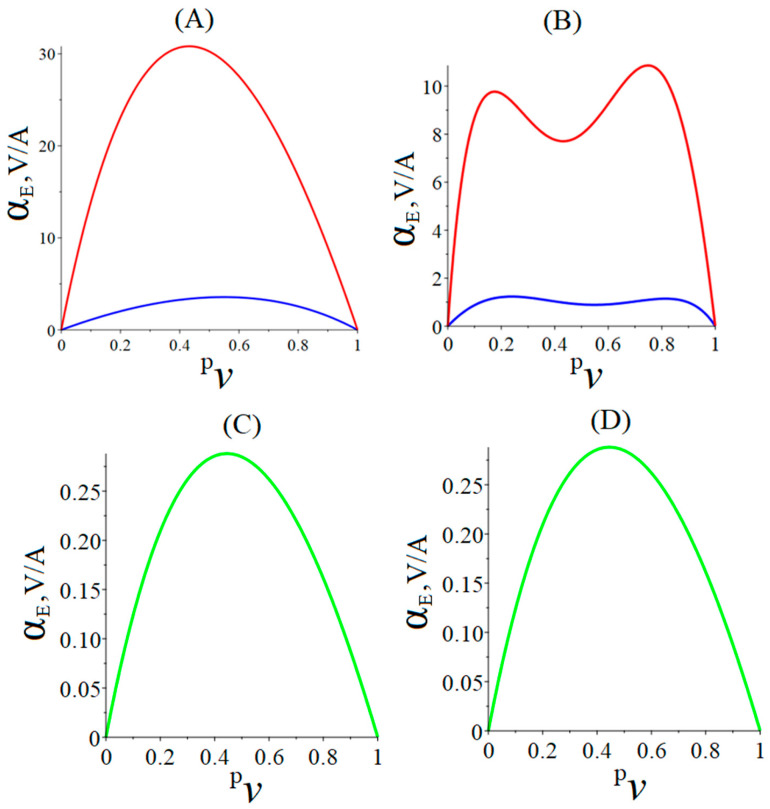
Dependence of ME voltage coefficient on the volume fraction of the piezoelectric phase of a ME composite in a quasi-static regime: longitudinal mode, symmetric ME composite (**A**), bending and longitudinal mode, asymmetric ME composite (**B**); longitudinal-shear mode, symmetric ME composite (**C**); torsional and longitudinal shear mode, asymmetric ME composite (**D**). Blue line is for PZT layer, red line is for LN cut y + 128° layer, green line for GaAs layer.

Taking into account Equation (65) and $D_{3}=0$, we obtain the transverse component of the electric field strength in the piezoelectric phase:

(68) Ep3=−h36(A−zB),

Then, electrical voltage in piezoelectric:

(69) U=∫z0−tpz0−h36A−zBdz=−h36Atp+12Btp2z0−tp,

Substituting the values found from the system of equations *A* and *B* in Equation (69), find the voltage on the piezoelectric and get the final expression for the ME voltage coefficient:

(70) αE=tptmGmq16h36[GpDtp3+Gmtm3+[Gm2tm2(b2+tm2)+GpDtp2(GpDtp2+6tm2Gm+4tmGmtp+b2Gp)++b2(Gptp+Gmtm)]+Gmtptm(b2Gp+4GpDtm2+GpDb2)](tp+tm),

It can be seen from Figure 3 that for each ME structure in the quasi-static regime, there is an optimal ratio of volume fractions between the phases of the composite at which the ME voltage coefficient increases to its maximum value. In the case of an asymmetric ME composite with bending deformation, the associated longitudinal deformation has different signs above and below the neutral plane. This causes such a specific behavior in the dependence of the ME voltage coefficient on the piezoelectric volume fraction. As we noted earlier, the magnitude of the ME stress coefficient for the torsional mode is an order of magnitude smaller than for the longitudinal-shear one. Therefore, the torsional mode almost does not contribute to the value of the ME voltage coefficient in the quasi-static regime, and the ME voltage coefficient in the quasi-static regime is mainly determined by the contribution of the longitudinal-shear mode. Therefore, the general nature of the dependence of the ME voltage coefficient on the volume fraction of the piezoelectric for the torsional mode remains the same as for longitudinal-shear mode.

This section provides an overview of several methods of analytical calculations of the direct ME effect in symmetric and asymmetric ME composites in the EMR region and in the quasi-static mode for various modes of ME composites. Expressions are obtained for the ME voltage coefficients in the quasi-static and EMR modes. A comparison of the obtained theoretical calculations of the ME voltage coefficient with known experimental data from previously published studies for various ME structures showed satisfactory agreement. The results obtained can be used to design ME composites and devices based on the ME effect in the low-frequency range. Next, it is planned to perform similar theoretical calculations of the inverse low-frequency ME effect and compare the results with experimental data.

### 2.2. Methods Based on Computer Programs/Software Products

Here we consider the works in which numerical methods of FEM were used in the study of ME composites in the low-frequency range. The bending mode of the ME effect in asymmetric ME structures with a gradient magnetostrictive phase of FeCuNbSiB/Ni/PZT and FeCuNbSiB/FeNi/PZT was studied based on a nonlinear model of magnetostriction [37]. Analytical calculation methods were mainly used, but the magnetic field in gradient magnetostrictive phases was calculated using Ansoft 11.0, a commercial FEM magnetic field simulation program. The results of such a calculation of the dependence of the ME voltage coefficient on a bias field corresponded quite accurately to the experimental data obtained. In ref. [33], the ME effect in layered FeCuNbSiB/Terfenol-D/PZT and Terfenol-D/PZT structures was considered. The calculation was made entirely using the commercial FEM software package, Comsol Multiphysics 6.0. The field dependences of the ME coefficient obtained using this simulation were in good agreement with the experimental data of other authors. It was shown that the FeCuNbSiB/Terfenol-D/PZT three-layer structure with a gradient magnetostrictive phase is self-magnetizing and has a rather strong ME effect at zero bias field. In ref. [38], the authors studied the ME effect in the Terfenol-D/PZT two-layer structure. For the calculation, they used their own programs based on independently developed 2D FEM algorithms. The field dependence of the ME coefficient with respect to voltage, obtained with the help of such a simulation, correlated relatively well with the experimental data. Simulations were also carried out at various temperatures and preliminary mechanical stresses. In ref. [40], a study of the ME antenna based on the magnetostrictive–piezoelectric structure of Terfenol-D/PZT was carried out. Using Comsol, the frequency dependence of the inverse ME coefficient was obtained, which is in good agreement with the experimental data of other authors. Moreover, with the help of Comsol, the directivity patterns of the near field of the ME antenna were obtained for several design options and several values of the bias field. Xu et al. [47] investigated a ME compass based on the Metglas/PZT magnetostrictive–piezoelectric structure. Basically, the authors used analytical methods of calculation. But to calculate the distribution of the magnetic field in the magnetostrictive phase, Comsol was used. Fedulov et al. [48] studied the ME effect in stripe and periodic ME structures based on a two-layer Ni/PZT magnetostrictive–piezoelectric composite. The authors partially used analytical calculation methods but also used Comsol to calculate the magnetic field, strain distribution, and resonant frequency of the circular mode. In [42], the ME effect in a two-layer CoFe_2_O_4_/PZT composite was studied. Here, the authors used their own program based on a self-developed hybrid/mixed FEM algorithm. The advantage of the developed hybrid/mixed FEM algorithm over particle FEM (PFEM) in the study of bending vibrations was shown. In ref. [43], a study was made on the static ME effect in asymmetric and symmetric ME structures of FeCo/PZT and FeCo/PZT/FeCo. The authors used their own software that implements a self-developed FEM algorithm. They also compared the solution obtained with the solution found using analytical methods and established a fairly close match between the indicated solutions. Based on a nonlinear model of magnetostriction, the authors of [38] studied the longitudinal mode of the ME effect in asymmetric ME structures with a gradient magnetostrictive phase of FeCuNbSiB/Ni/PZT and FeCuNbSiB/FeNi/PZT in the EMR mode. The equivalent circuit method was mainly used, but the magnetic field in gradient magnetostrictive phases was calculated using Ansoft. The results of calculating the field dependence of the ME coefficient for voltage were in good agreement with the experimental data. In [41], the bending and longitudinal modes of the ME effect in the asymmetric Terfenol-D/PZT structure and the symmetric Terfenol-D/PZT/Terfenol-D structure were considered in the quasi-static and EMR ranges. To find the ME voltage coefficient, the authors used their own program based on an independently developed algorithm for two-dimensional FEM using the Galerkin method. The resulting field and frequency dependences of the ME voltage coefficient were in good agreement with the experimental data obtained by other authors. In ref. [49], Spetzler et al. studied the ΔE-effect in the AlN/poly-Si/(Fe_90_Co_10_)_78_Si_12_B_10_ structure in the bending and torsional modes. The authors used Comsol and obtained 3D transverse displacement plots for fundamental and higher electromechanical resonance modes. Also in the work, the dependences of the normalized resonant frequencies on the magnitude of the magnetic field were obtained. In ref. [50], Hähnlein et al. investigated the ΔE-effect of the TiN/AlN/Ni structure in the bending mode. Using Comsol, the authors obtained the frequency dependence of magnetic sensitivity on the thickness of the Ni layer for its various crystallographic orientations. It was shown that the results of the FEM simulation are closer to the experimental data than the results of analytical calculations using the Euler–Bernoulli theory. In ref. [45], the authors developed a model of an ME magnetic field sensor based on the CoFe_2_O_4_/PZT structure and a tunable ME inductor based on the Metglas 2605CO/PZT/Metglas 2605CO symmetrical structure. When modeling, they used their own software for two-dimensional FEM according to the Galerkin method with the discretization of nodal elements. The frequency dependence of the ME coefficient calculated in this way showed an increase in the ME effect at EMR in accordance with the experimental data. In ref. [46], for the ME structure of Terfernol-D/PZT/Terfernol-D, the longitudinal mode of the ME effect in the EMR region was studied in order to develop an ME harvester for medical applications. For 2D FEM, the authors used their own software and obtained the frequency dependence of the ME voltage coefficient and the dependence of power on the value of the payload resistance for four different cases obtained with the orientations of the magnetization and the polarization in the composite. It was shown that the maximum power of the ME harvester was obtained by using the L-T mode of the ME effect. In ref. [50], the static ME effect in magnetostrictive–piezoelectric structures CoFe_2_O_4_/PZT with 1–3 and 0–3 connectivity was described using Comsol. The distributions of the electric potential, mechanical strain, and magnetization were obtained. For connectivity 0–3, the Monte Carlo method was used to take into account the possibility of various configurations of sizes and arrangements of CoFe_2_O_4_ inclusions in the PZT matrix at a constant volume fraction of CoFe_2_O_4_. The obtained field dependences of the ME voltage coefficient agreed quite well with the experimental data of other authors. Thus, today, the software for FEM is already widely used in the study of the ME effect in the low-frequency range. The results of such modeling are in fairly good agreement with the results of calculations by analytical methods, when this can be verified. It is important to note that the results of FEM calculations compared with experimental data showed good agreement.

## 3. High-Frequency Range

### 3.1. Analytical Methods

Recently, the magnetoelectric (ME) effect in the microwave range has been actively investigated. This is due to the fact that ME composites have a large number of advantages, and thus they open up great technological prospects for new multifunctional devices. One of the methods of studying the ME effect in the field of high frequencies is currently the analytical method, which is still used in many scientific papers, in parallel, for example, with a method based on the use of computer programs. Next, the analytical methods used to study the ME effect in the microwave range will be considered in more detail. A method for solving a number of research and applied problems using the layered structures YIG/PZTtBS-2, YIG/PZTT-19, and YIG/PZTtBS-1 was presented [1]. Based on this study, it became possible to obtain the necessary data to solve the problem of optimizing the structure of composite heterophase ME materials. In ref. [52], a theoretical approach was developed for the study of the ME effect in two-phase composites. The calculation of the generalized ME sensitivity tensor is given, and its estimates at low and high frequencies are given. The presented results can be used to calculate microwave devices of the magnetic type with electric control. Two models were discussed [53]: A simple two-layer (bimorph) NFO/PZT structure and a multilayer structure, which was considered a homogeneous medium. For the NFO/PZT structure, the theory predicted a five times stronger effect than in YIG/PZT. The expressions were obtained for the structures LFO/PZT, NFO/PZT, and YIG/PZT, linking the components of the magnetic susceptibility tensor and the ME sensitivity of the composite with the ME coupling constants [1]. Calculations showed the greatest effect of the electric field for NFO/PZT and the weakest effect for YIG/PZT. In refs. [54,55], a theoretical model of the microwave ME effect in two-layer composites (111) YIG/(001) PMN-PT was presented. The obtained theoretical model showed that the ME coupling in two-layer composites is an order of magnitude stronger than in polycrystalline composites, and the coupling strength depends on the orientation of the magnetic field and is higher for out-of-plane H than for in-plane H. Moreover, the results obtained were used to design and evaluate the performance characteristics of the filter and phase shifter, tunable by an electric field. For a filter consisting of ME resonators, the estimated insertion loss was only 1.1 dB. In the review [20], a filter based on a layered YIG/PZT structure was considered. It can be tuned to 2% of the central frequency with a nominal electric field of 3 kV/cm and the possibility of implementing a passive phase shifter tuned by an electric field based on the YIG/PMN-PT structure. The authors of [56] have presented the results of theoretical and experimental studies of an attenuator, a bandpass filter, and a phase shifter based on YIG/PMN-FET and YIG/PZT layered structures under the action of an external electric field up to 8 kV/cm. The insertion loss of the attenuator varied from 26 dB to 2 dB at 7251 MHz; the band-pass filter tuning range of 25 MHz at a frequency of 7360 MHz was obtained; and in the frequency range of 6–9 GHz, a maximum phase shift of 30–40 degrees was obtained. In ref. [57], progress on magnetic field-tunable RF/microwave devices is covered, including novel non-reciprocal tunable bandpass filters with ultra-wideband isolation, compact, low-loss, high-power-handling phase shifters, etc. These novel tunable multiferroic heterostructures and devices and tunable magnetic devices provide great opportunities for next-generation reconfigurable RF/microwave communication systems and radars, spintronics, magnetic field sensing, etc. In ref. [58], a model of the resonant RLC circuit and an ideal transformer model based on ME elements were created, which were used to calculate the microwave resonator and filter. It is found that the model can effectively predict the center frequency and bandwidth for the resonator, and the use of layered ME composites in filters allows not only to expand the bandwidth but also to control the operating frequency band by adjusting external electrostatic and magnetostatic fields. Nan et al. [59] quantified the coexistence of deformation and surface-charge ME bonding at the ultrathin interface of Ni_0.79_Fe_0.21_/PMN-PT and Ni_0.79_Fe_0.21_/Cu/PMN-PT heterostructures. The heterostructure N_i0.79_Fe_0.21_/PMN-PT demonstrates a high-voltage-induced change in the effective magnetic field of 375 Oe, enhanced by a surface charge at the PMN-PT interface; by introducing a Cu layer at the PMN-PT interface, the change in the electric field of the effective magnetic field was 202 Oe. Xue et al. [60] demonstrated the control of the electric field by the effect of two-magnon scattering excited by the rotation of the lattice in the plane of a multiferroic heterostructure LSMO/(011) PMN-PT. The effect of two-magnon scattering demonstrates strong anisotropic and temperature-dependent behavior due to the angular dependence of FMR measurements. A large modulation of the electric field of magnetic anisotropy (464 Oe) and the FMR line width (401 Oe) is achieved at 173 K. Hou et al. [61] obtained multiferroic heterostructures by growing MnZn ferrite films on ferroelectric substrates using pulsed laser deposition technology. Enhanced ME coupling was achieved at critical angles of two-magnon scattering when the FMR field is shifted by −653 and 211 Oe. It is shown that the contribution of the effects of two-magnon scattering is much greater than the contribution of the usual deformation effect. The article [62] was devoted to microwave losses in thin films of YIG, MnZn–ferrite and NiZn–ferrite deposited by pulsed laser deposition, with thicknesses of 1.8, 0.5, and 1.5 mm, respectively. The FMR field, depending on the angle of the field, in this work, confirmed the assumption of a homogeneous mode response. Duan et al. [63] provided a study of the surface ME effect using density functional calculations for ferromagnetic films of Fe (001), Ni (001), and Co (0001) in the presence of an external electric field. It is found that the magnitude and sign of the surface ME coefficient depend on the density and spin polarization of charge carriers near the Fermi level of a ferromagnetic metal film. In ref. [61] a high field shift of the electrostatically tunable FMR up to 600 Oe is observed in heterostructure Fe_3_O_4_/PMN-PT, corresponding to a large ME coefficient of 67 Oe∙cm/kV. The Fe_3_O_4_/PZN-PT heterostructure demonstrated a record-high electrostatically tunable FMR field range of 860 Oe with a line width of 330–380 Oe, corresponding to the ME coefficient of 108 Oe∙cm/kV. The static ME interaction has also been investigated, and the maximum change in the squareness ratio induced by the electric field is observed at 40% in Fe_3_O_4_/PZN-PT. Lou et al. in [65] have presented a study of multiferroic heterostructures of FeGaB/PZN-PT. It was shown that such a heterostructure has a large average ME coupling coefficient of 94 Oe∙cm/kV and a giant ME coupling coefficient of 2365 Oe∙cm/kV in the region of the electric field-induced phase transition PZN-PT. The heterostructures also demonstrated a wide frequency range of FMR tunable by an electric field, from 1.75 to 7.57 GHz at zero bias field. Tatarenko et al. [66] have considered the analytical dependences of FMR line shifts on the electric field strength for two-layer ME composites based on piezoelectric plates of PZT, PMN-PT, and PZN-PT and ferromagnetic thin films of Ni, Fe, and Co. The results of the study of the microwave ME effect for structures consisting of various ferromagnetic metals (FM) and piezoelectrics were considered more detailed [67]. The following materials were used as FM: Ni, Fe, Co, NiFe, and FeGaB, and as piezoelectrics: quartz, PZT, PMN–PT, and PZN-PT. Moreover, in this article, the “substrate effect” in the piezoelectric/YIG/GGG ME structure was discussed.

#### 3.1.1. ME Effect in Ferromagnetic Metal—Piezoelectric

**Figure 4:**
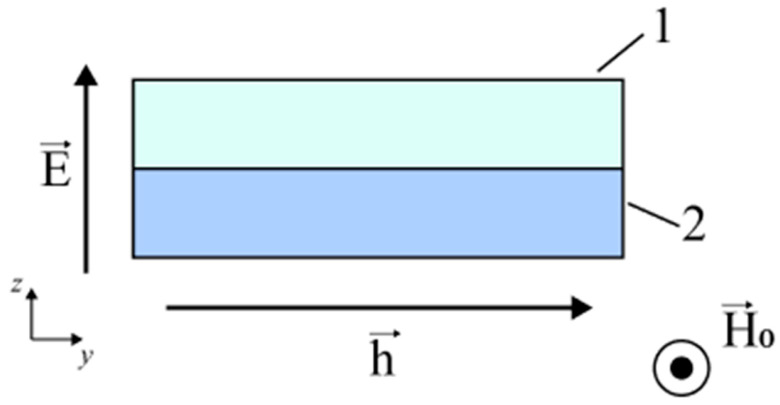
Two-layer ME structure: 1—magnetic component, 2—piezoelectric. $\vec{E}$, $\vec{H_{0}}$, $\vec{h}$ directions of the bias field, high-frequency magnetic field and electric field, respectively.

The authors present a methodology for calculating the FMR line shift during the microwave ME effect in two-layer structures and, as a result, draw appropriate conclusions and recommendations on the selection of materials to achieve the greatest microwave ME effect. The structure shown in Figure 4 is considered, where 1 is the magnetic component and 2 is piezoelectric. Figure 4 also shows the directions of the magnetizing and alternating magnetic fields.

For the ME composite, the orientation of the piezoelectric phase corresponds to the crystallographic direction [011] for PMN-PT and PZN-PT. Moreover, for PMN-PT, the crystallographic directions [100] and [1,1] coincide with the *x* and *y* axes, respectively.

The Landau–Lifshitz–Hilbert equation is used for a thin nickel film in a high-frequency magnetic field and in the presence of a bias field *H*_0_ under the condition $h\ll H_{0}$ and taking into account dissipation, which has the form:

(71) $$\frac{\partial \vec{M}}{\partial t}=\gamma \left[\vec{M}\times \frac{\partial W}{\mu_{0}\partial \vec{M}}\right]+i\omega \alpha \frac{\left[{\vec{M}}_{0}\times \vec{m}\right]}{M_{0}},$$

where the full magnetization is determined by the sum of the equilibrium *M*_0_ and high-frequency *m* components:

(72) $$\vec{M}={\vec{M}}_{0}+\vec{m},$$

where the free energy density is determined by the formula:

(73) $$W=-\mu_{0}\vec{M}\cdot \vec{H}+\frac{\mu_{0}}{2}\sum N_{11}^{i}M_{1}^{2}+\frac{\mu_{0}}{2}\sum N_{22}^{i}M_{2}^{2}+\frac{\mu_{0}}{2}\sum N_{33}^{i}M_{3}^{2},$$

where γ is the gyromagnetic ratio, µ_0_ is the magnetic constant, ω is the frequency of harmonic oscillations, *N*_kk_^i^ is the demagnetizing factor, and α is the dissipation parameter.

To solve Equation (71), the method of linearization by a small parameter is used, and the system of three equations with three unknown variables is obtained. Solving this system of equations by Cramer’s rule, we find the tensor of high-frequency magnetic susceptibility that connects the components of high-frequency magnetization with the components of a high-frequency magnetic field. Then, the imaginary parts of the complex components for the tensor of high-frequency magnetic susceptibility ${\chi^{\prime \prime }}_{22}$ and ${\chi^{\prime \prime }}_{33}$ were found:

(74) $$\begin{array}{l} {\chi^{\prime \prime }}_{22}=-\alpha \gamma M_{0}\omega \frac{\omega^{2}\left(1+\alpha^{2}\right)-\omega_{0}^{2}+\gamma^{2}\left(H_{0}+\left\{\sum N_{33}^{i}-\sum N_{11}^{i}\right\}M_{0}\right)\left(2H_{0}+\left\{\sum N_{22}^{i}+\sum N_{33}^{i}-2\sum N_{11}^{i}\right\}M_{0}\right)}{{\left[\omega^{2}\left(1+\alpha^{2}\right)-\omega_{0}^{2}\right]}^{2}+\gamma^{2}\alpha^{2}\omega^{2}{\left(2H_{0}+\left\{\sum N_{22}^{i}+\sum N_{33}^{i}-2\sum N_{11}^{i}\right\}M_{0}\right)}^{2}}\\ {\chi^{\prime \prime }}_{33}=-\alpha \gamma M_{0}\omega \frac{\omega^{2}\left(1+\alpha^{2}\right)-\omega_{0}^{2}+\gamma^{2}\left(H_{0}+\left\{\sum N_{22}^{i}-\sum N_{11}^{i}\right\}M_{0}\right)\left(2H_{0}+\left\{\sum N_{22}^{i}+\sum N_{33}^{i}-2\sum N_{11}^{i}\right\}M_{0}\right)}{{\left[\omega^{2}\left(1+\alpha^{2}\right)-\omega_{0}^{2}\right]}^{2}+\gamma^{2}\alpha^{2}\omega^{2}{\left(2H_{0}+\left\{\sum N_{22}^{i}+\sum N_{33}^{i}-2\sum N_{11}^{i}\right\}M_{0}\right)}^{2}} \end{array}$$

where:

(75) $$\omega_{0}^{2}=\gamma^{2}\left(H_{0}+\left\{\sum N_{22}^{i}-\sum N_{11}^{i}\right\}M_{0}\right)\left(H_{0}+\left\{\sum N_{33}^{i}-\sum N_{11}^{i}\right\}M_{0}\right),$$

The resonant values of the bias field for ${\chi^{\prime \prime }}_{22}$ and ${\chi^{\prime \prime }}_{33}$, found numerically in Maple for the frequency *f* = 10 GHz are consistent and also coincide with the value that was found from the following resonance condition, with an accuracy of 1 A/m:

(76) $$\omega^{2}=\omega_{0}^{2},$$

Using Equation (75), Equation (76) is represented as a square with respect to *H*_0_ and solved:

(77) $$\gamma^{2}H_{0}^{2}+\gamma^{2}M_{0}\left(\sum N_{22}^{i}+\sum N_{33}^{i}-2\sum N_{11}^{i}\right)H_{0}-\omega^{2}=0,$$

(78) $$H_{0}=\frac{\gamma M_{0}\left(2\sum N_{11}^{i}-\sum N_{22}^{i}-\sum N_{33}^{i}\right)+\sqrt{\gamma^{2}M_{0}^{2}{\left(\sum N_{22}^{i}-\sum N_{33}^{i}\right)}^{2}+4\omega^{2}}}{2\gamma },$$

Then the following equation is derived to determine the shift of the FMR line in a two-layer ME structure, FM/piezoelectric:

(79) δHE=−3λ100Q2Tm1−Tm2+Q3Tm1μ0M0Q1,

where:

(80) $$\begin{array}{l} Q_{1}=2H_{0}+M_{0}+2H_{a}\\ Q_{2}=H_{0}+M_{0}+H_{a}\\ Q_{3}=H_{0}+H_{a} \end{array},$$

(81) Tm1=d31sm11tp+d31sp22tm−d32sm12tp−d32sp12tmtpEsm112tp2+sm11sp11tmtp+sm11sp22tmtp−sm122tp2−2sm12sp12tmtp+sp11sp22tm2−sp122tm2Tm2=d32sm11tp+d32sp11tm−d31sm12tp−d31sp12tmtpEsm112tp2+sm11sp11tmtp+sm11sp22tmtp−sm122tp2−2sm12sp12tmtp+sp11sp22tm2−sp122tm2,

and λ_100_—magnetostrictive coefficient.

**Figure 5:**
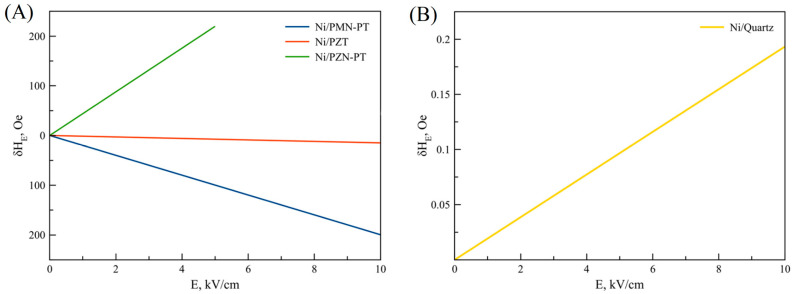
Theoretical dependences for FMR line shift on electric field strength for (**A**) Ni/PMN-PT, Ni/PZT, Ni/PZN-PT; (**B**) Ni/quartz ME structures.

Figure 5 shows the obtained theoretical dependences for the FMR line shift on the electric field for some ME structures.

Ferromagnetic metals are characterized by the skin effect, which manifests itself in the fact that the magnitude of the magnetic field decreases as the distance inside the ferromagnetic metal increases from its surface. Therefore, thin films of ferromagnetic metals with a thickness of no more than a few tens of nm are used in practice. In the above calculation, the skin effect is not taken into account. This may lead to some discrepancies with experimental data. However these are small, since the film thickness of the ferromagnetic metal is small.

The equations used to describe the FMR in the magnetostrictive phase are three-dimensional. The equations of mechanics do not take into account bending deformations and are therefore two-dimensional, taking into account only measurements of length and width. If the thickness of the magnetostrictive phase were comparable to the thickness of the piezoelectric phase, then it would be necessary to take into account bending deformations and use three-dimensional mechanical equations that also take into account the thickness measurement.

The neglect of bending deformations in the calculation can also potentially lead to some discrepancies with the experimental data. However again, these are small, since the thickness of the ferromagnetic metal film is small compared to the thickness of the piezoelectric phase. Therefore, the ME composite is almost symmetrical, and the effect of bending deformations is very small. Another assumption that we make in the calculations is that we believe that the magnetizing constant field is sufficiently large, so the magnetostrictive phase is a single-domain region, and the direction of the equilibrium magnetization coincides with the direction of the magnetizing field. Due to the use of this assumption, there may also be some discrepancies with the experimental data. But at the used microwave frequencies of the order of several GHz, the necessary magnetizing field is indeed quite large. Therefore, this reason should not lead to a big error.

As can be seen from Equation (79), the shift of the FMR line is quite difficult to depend on the corresponding components ^m^*T*_1_ and ^m^*T*_2_, which also quite non-trivially depend on the components of the piezoelectric tensors *d*_31_ and *d*_32,_ respectively. As a result, it can be concluded that the shift sign of the FMR line, taking into account the impact of all the previously listed factors, can be both positive and negative.

#### 3.1.2. ME Effect in Ferrite—Piezoelectric

Then a method is given for calculating the FMR line shift during the microwave ME effect in a two-layer ME composite with a thin film of YIG ferrite used as the magnetostrictive phase of the composite, and recommendations are given on the choice of piezoelectrics to observe the maximum microwave ME effect.

Observation of the microwave ME effect consists of measuring the FMR line shift when exposed to a constant electric field. Of all the magnetic materials used for FMR observation, YIG has one of the smallest FMR resonance line widths. Therefore, in practice, YIG-based composites are the most convenient for observing the microwave ME effect.

A thin plate YIG (001) is considered, in the plane of which a bias field *H*_0_ is applied. Suppose the magnitude of this permanent field is large enough and the YIG plate is magnetized to saturation. The direction of the magnetic field *H*_0_ coincides with the axis 2 (*y*) and has components (0, *H*_0_, 0); also, the equilibrium magnetization has components (0, *M*_0_, 0). The crystallographic direction [100] of the YIG plate coincides with the 1 (*x*) axis and is directed along the width of the YIG plate. The case is considered for the bias field *H*_0_ directed perpendicular to the plane of the YIG plate. In this case, the direction of the magnetic field *H*_0_ coincides with the axis 3 (*z*) and has components (0, 0, *H*_0_), as well as a component of equilibrium magnetization: (0, 0, *M*_0_). Then, the necessary imaginary parts of the complex components for high-frequency magnetic susceptibility ${\chi^{\prime \prime }}_{11}$ and ${\chi^{\prime \prime }}_{33}$ were determined.

For the bias field *H*_0_, the resonant values for ${\chi^{\prime \prime }}_{11}$, ${\chi^{\prime \prime }}_{33}$ found numerically in Maple for the frequency *f* = 10 GHz are consistent and also coincide with the value that was found from the following resonance condition $\omega^{2}=\omega_{0}^{2}$, where:

(82) $$\omega_{0}^{2}=\gamma^{2}\left(H_{0}+\left\{\sum N_{11}^{i}-\sum N_{22}^{i}\right\}M_{0}\right)\left(H_{0}+\left\{\sum N_{33}^{i}-\sum N_{22}^{i}\right\}M_{0}\right),$$

The equations for determining the magnitude of the bias field at orientation along the axes 2 (*y*) and 3 (*z*) have the following form:

(83) $$H_{0}=\frac{\gamma M_{0}\left(2\sum N_{22}^{i}-\sum N_{11}^{i}-\sum N_{33}^{i}\right)+\sqrt{\gamma^{2}M_{0}^{2}{\left(\sum N_{11}^{i}-\sum N_{33}^{i}\right)}^{2}+4\omega^{2}}}{2\gamma },$$

(84) $$H_{0}=\frac{\gamma M_{0}\left(2\sum N_{33}^{i}-\sum N_{11}^{i}-\sum N_{22}^{i}\right)+\sqrt{\gamma^{2}M_{0}^{2}{\left(\sum N_{11}^{i}-\sum N_{22}^{i}\right)}^{2}+4\omega^{2}}}{2\gamma },$$

Next, the FMR line shift equations are obtained in a two-layer magnetoelectric structure, YIG/piezoelectric, with the orientation of the bias field along the 2 (*y*) axis and 3 (*z*) axis, respectively:

(85) δ1HE=3λ100Q1Tm1−Tm2−Q3Tm2μ0M0Q2,

(86) δ2HE=3λ100Tm1+Tm22μ0M0,

where:

(87) $$\begin{array}{l} Q_{1}=H_{0}-H_{a}+M_{0}\\ Q_{2}=2\left(H_{0}-H_{a}\right)+M_{0}\\ Q_{3}=H_{0}-H_{a} \end{array},$$

**Figure 6:**
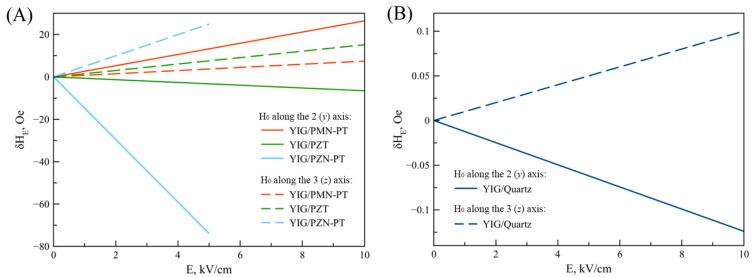
Theoretical dependences for FMR line shift on electric field strength in the ME composites: (**A**) YIG/PMN-PT, YIG/PZT, YIG/PZN-PT; (**B**) YIG/quartz. The solid line is the FMR line shift with the bias field directed along the 2 (*y*) axis in the plane of the plate, and the dotted line is the FMR line shift with the bias field directed along the 3 (*z*) axis perpendicular to the YIG plate.

Figure 6 shows the obtained theoretical dependences for the FMR line shift on the electric field for some ME structures.

Based on the graphs shown in Figure 6, the authors draw the following conclusions. Firstly, the greatest FMR line shift was determined for the ME composite YIG/PZN-PT for a bias field oriented along the 2 (*y*) axis and at an electric field strength of up to 5 kV/cm. In addition, high values of the FMR line shift were determined with the bias field oriented along the 2 (*y*) axis for YIG/PMN-PT composites. This is because the piezoelectric coefficients of PZN-PT and PMN-PT are quite high, and the lateral faces (011) of PMN-PT and PZN-PT crystals coincide with the crystallographic directions [100] and [1,1]. For this case, the value (*d*_32_-*d*_31_) has the maximum value and is proportional to the FMR line shift. Secondly, due to the fact that the piezoelectric coefficients of PZT are less than those of PMN-PT, the shift of the FMR line for a ME composite with PZT is less than for a composite with PMN-PT, and also, due to the symmetry of PZT, its values *d*_31_ and *d*_32_ are the same. Because quartz and langatate have piezoelectric coefficients about two orders of magnitude lower than those of PZT and PMN-PT, the FMR line shift in the electric field for ME composites with quartz and langatate has a low value. As a result, due to the fact that the width of the FMR line for YIG is about 1 Oe, the FMR line shift in the electric field in the ME composites under consideration can be observed experimentally.

#### 3.1.3. ME Effect in Piezoelectric/YIG/GGG

**Figure 7:**
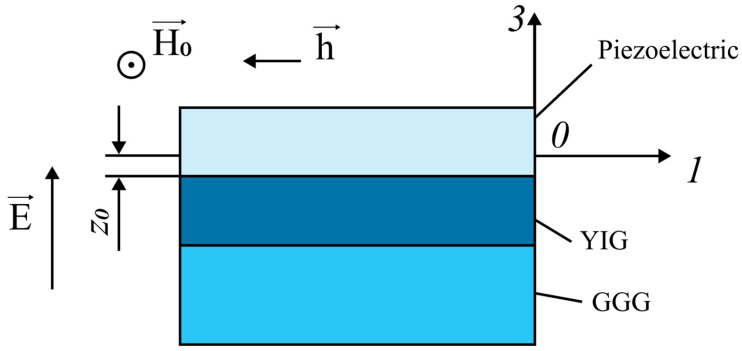
Structure of the ME composite piezoelectric/YIG/GGG. $\vec{E}$, $\vec{H_{0}}$, $\vec{h}$–directions of the bias field, high-frequency magnetic field and electric field, respectively.

In the last paragraph, it is considered how the GGG substrate reduces the FMR line shift for ME structures (piezoelectric/YIG/GGG) shown in Figure 7.

Suppose the magnitude of the bias field *H*_0_ is large enough and the YIG plate is magnetized to saturation. The direction of the magnetic field *H*_0_ coincides with the axis 2 (*y*) and has components (0, *H*_0_, 0); also, the equilibrium magnetization has components (0, *M*_0_, 0). The crystallographic direction [100] of the YIG plate coincides with the 1(*x*) axis and is directed along the width of the YIG plate.

The components of the strain tensor along axes 1 and 2, since the longitudinal and bending modes of deformation are excited along the X and Y axes in an asymmetric ME structure:

(88) $$S_{1}=A_{1}+zB_{1},$$

(89) $$S_{2}=A_{2}+zB_{2},$$

where the longitudinal mode is associated with unknown constants *A*_1_ and *A*_2_, and the bending mode is associated with unknown constants *B*_1_ and *B*_2_. They are found from the solution of a system of four linear inhomogeneous equations, which is described in detail in the paper.

The stress components of the magnetostrictive phase, which now take into account the influence of the substrate and bending vibrations, are determined by the following equations:

(90) Tm1=cm11S1+cm12S2Tm2=cm11S2+cm12S1,

The stress components of the magnetostrictive phase of the ME composite depend on *z*, but because its thickness is small, the coordinate of the middle of this phase is taken as *z* as:

(91) z=−tm2−z0,

where:

(92) z0=tp2cp12E−tm2cm12−tscs122tm+ts2tpcp12E+tmcm12+tscs12,

The stress components found in this way are YIG ^m^*T*_1_ and ^m^*T*_2_, which are substituted into Equation (85), which gives the FMR line shift for the direction of the magnetic field *H*_0_, which coincides with the axis 2 (*y*). Similarly, to calculate the FMR line shift when the direction of the magnetic field *H*_0_ coincides with the axis 3 (*z*), the components found for YIG mechanical stresses ^m^*T*_1_ and ^m^*T*_2_ are substituted into Equation (86).

**Figure 8:**
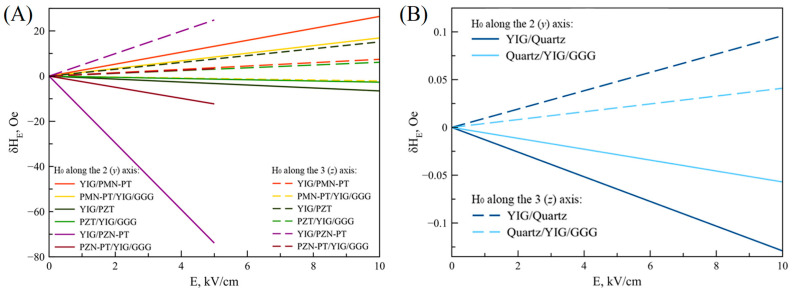
Theoretical dependences for FMR line shift on electric field strength for ME structures: (**A**) YIG/PMN-PT, PMN-PT/YIG/GGG, YIG/PZT, PZT/YIG/GGG, YIG/PZN-PT, PZN-PT/YIG/GGG; (**B**) YIG/quartz, quartz/YIG/GGG. The solid line is the FMR line shift with the bias field directed along the 2 (*y*) axis in the plane of the plate, and the dotted line is the FMR line shift with the bias field directed along the 3 (*z*) axis perpendicular to the YIG plate.

Then, graphs of the FMR line shift on the applied electric field strength to the ME composite piezoelectric/YIG/GGG are given for the following orientations of the bias field: The bias field directed along the 2 (*y*) axis in the plane of the plate, and the bias field directed along the 3 (*z*) axis perpendicular to the YIG plate. For comparison, Figure 8 shows the corresponding dependences for the ME composite piezoelectric/YIG.

Based on the dependencies and comparisons, the following conclusions can be drawn. Firstly, in the presence of a GGG substrate, the FMR line shift is on average two times less for ME composites piezoelectric/YIG/GGG than for composites YIG/piezoelectric for the two bias field orientations considered. Without the GGG substrate, the FMR line shift is approximately seven times greater for ME composite YIG/PZN-PT than for composite PZN-PT/YIG/GGG. In addition, for the ME composite PMN-PT/YIG, in the presence of a GGG substrate, the sign of the FMR line shift changes at the bias field directed along the 3 (*z*) axis perpendicular to the YIG plate. The results of theoretical calculations demonstrate the “substrate effect”. The “substrate effect” is explained by the fact that mechanical deformations arising in a piezoelectric from an external electric field in the presence of a GGG substrate are transmitted both to the magnetostrictive phase and to the substrate. This effect leads to a decrease in the FMR line shift due to a decrease in mechanical stresses in YIG compared to the ME composite without a substrate.

The analytical methods given in this section allow us to obtain theoretical dependences of the FMR line shift on the electric field in different structures, thereby predicting the possibility of experimentally observing this FMR line shift in an electric field.

In addition, these methods help to draw conclusions about increasing the effectiveness of the ME effect. For example, to reduce the weight and size parameters of the ME devices, it is proposed to reduce the magnitude of the bias field. A possible option for creating a bias field inside the ME composite may be the use of gradient ME composites, the magnetostrictive phase of which is formed from one layer of soft magnetic material and one layer of hard magnetic material. With a certain thickness of the layers of the magnetostrictive phase of the ME composite, a certain permanent magnetic field can be created. To reduce the control electric field strength magnitude, the materials of the piezoelectric phase of composites must have high piezoelectric coefficients. For example, for ME composites with PZN-PT and PMN-PT as piezoelectrics, at the same magnitude of the FMR line shift during the microwave ME effect, a low value of the control electric field is observed compared to ME composites with other piezoelectrics. Thanks to the analytical methods considered, it can be concluded that the presence of a passive substrate in the ME composite significantly reduces the magnitude of the FMR line shift during the microwave ME effect. It is necessary to reduce the thickness of the substrate and use a less mechanically rigid material as a substrate to reduce the negative effect of the substrate on the FMR line shift. The detailed calculations of the FMR line shift taking into account the “substrate effect” in the electric field in the ME composites under consideration confirm a significant decrease in the FMR line shift for ME composites piezoelectric/YIG/GGG compared with ME composites piezoelectric/YIG. In the presence of a substrate, the FMR line shift for ME composites is on average two times less than for composites without a substrate due to the transfer of mechanical deformations from the piezoelectric to both the YIG layer and the GGG substrate.

Thus, analytical methods can be used in the design of efficient microwave ME devices.

### 3.2. Methods Based on Computer Programs/Software Products

Here we consider the works in which numerical methods of FEM were used in the study of ME composites in the microwave range.

In ref. [68], the authors explore the application of the YIG/GGG/PZT layered structure for designing microwave ME devices based on a slot line, a microstrip line, and a coplanar waveguide. To calculate the frequency dependence of S-parameters, Ansoft’s commercial High-Frequency System Simulator (HFSS) 15.0 software for electromagnetic FEM was used. A relatively good agreement between the results of this simulation and experimental data on a coplanar waveguide was demonstrated. In ref. [70], V. Lobekin et al. carried out a computer simulation of the microwave ME valve. The device is based on the use of a YIG/GGG/PZT layered structure together with a coplanar line on a FLAN dielectric substrate. For modeling, the authors use HFSS. The frequency dependences of the S-parameters are found, and the prospects of the proposed design of the microwave valve are shown.

In ref. [69], the authors study a microwave ME antenna based on the FeGaB/AlN magnetostrictive–piezoelectric structure. The near-field simulation is performed in the commercial software Comsol Multiphysics 6.0. The commercial CST Studio 2022 software is used to model far-field characteristics such as gain and radiation pattern. The advantages of the investigated ME antenna are shown in comparison with the equivalent microloop magnetic antenna. The simulation results are in good agreement with the experimental data. In ref. [71], F. Rasoanoavy et al. considerd the design of a ME-tunable microstrip line based on a symmetric magnetostrictive–piezoelectric structure of CoFeB/PVDF/CoFeB. To simulate the microwave ME effect, the authors used Comsol. The simulation results are in good agreement with the experimental data. The review of works by various authors has shown that software products for numerical simulation are successfully used in modeling the ME effect in the high-frequency range.

The general theory of ME composites described above is applied in practice in the design of current sensors, magnetic field sensors, harvesters, etc. Moreover, these theoretical relationships are valid for any ME device in a wide frequency range.

## 4. Discussion

Along with the low-frequency direct ME effect described in detail in this article, the ΔE-effect, that is, the change in the elastic properties of the magnetostrictive phase caused by a magnetic field, is also very interesting and promising. ΔE-effect is the influence of a magnetic field on the mechanical deformation of unsaturated magnetic materials with magnetostriction [72]. This influence manifests itself in a magnetoelastic change in the direction of the magnetization vector in such materials. Adding a magnetostrictive strain to an elastic strain causes a change in the elastic coefficients. The dependence of the elastic coefficients on the magnitude of the magnetic field is used to measure this magnitude. The principle of operation of magnetic field sensors based on the ΔE-effect is as follows. The effect of a magnetic field on the magnitude of the elasticity coefficients causes a change in the resonant frequency in electromechanical bulk [73] and cantilever resonators [74]. Mechanical excitation and obtaining an output electrical signal are carried out using a piezoelectric layer. To excite mechanical vibrations, an alternating electrical voltage is applied to the piezoelectric at the frequency of the mechanical resonance of the system. The magnetic operating point is chosen near the value of the magnetic field strength at which the change of electromechanical resonance frequency caused by the magnetic field is maximum. The change in the resonant frequency determines the magnitude of the magnetic field. It is possible to use and detect several mechanical modes simultaneously [75]. Then the sensitivity of the magnetic field sensors using the ΔE-effect can increase. Another method to increase the sensitivity of such magnetic field sensors is the use of acoustic surface shear waves in the delay line sensor [76]. This method is based on the dependence of the transmitted signal delay on the magnitude of the magnetic field.

Recently, the possible use of ME magnetic field sensors for detecting magnetic signals from the heart and brain has been actively investigated [77,78,79]. These signals are magnetic fields of low amplitude and low frequency [80,81]. In magnetic field sensors based on the direct ME effect, it is possible to detect magnetic fields of such a small value only at high frequencies and with a small signal bandwidth of several Hz. Magnetic field sensors based on the ΔE-effect overcome these limitations and are well suited for applications in biomedicine [49,82]. Another important area of study of ME processes is the inverse ME effect, which consists of the appearance of magnetization in the magnetostrictive phase when an electric field is applied to the piezoelectric phase [32,83,84]. To measure the variable magnetic induction that appeared in the magnetostrictive phase, a coil with a wire wound on an ME composite is used. With a harmonic change in the magnetic induction on the coil due to the phenomenon of electromagnetic induction, an electrical voltage arises. By measuring this output voltage, one can find the experimental value of the inverse ME coefficient. Recently, the interest of researchers in the inverse ME effect has greatly increased. This is due to the fact that it is used for excitation in a transmitting low-frequency ME antenna [85,86,87]. Under the action of an alternating low-frequency electric field at the EMR frequency, mechanical vibrations are excited in the piezoelectric phase. Since the piezoelectric and magnetostrictive phases are mechanically connected, the mechanical vibrations from the piezoelectric phase pass into the magnetostrictive phase. This leads to the appearance of a variable magnetization in the magnetostrictive phase. Accordingly, this causes the emission of an electromagnetic field of the same frequency. Low-frequency ME antennas have significantly smaller dimensions and power consumption compared to traditional analogs. They can be used for underground and underwater communications. This review mainly describes the works in which the authors studied the microwave ME effect with a magnetizing field oriented along the main crystallographic axes of the magnetostrictive phase. A review of several papers was also made, where a very interesting case was studied when the bias field is directed arbitrarily in the plane of the magnetostrictive phase. Previously, the microwave ME effect was measured with a bias field directed along the principal axes of the magnetostrictive phase. Recent works, in which the angular dependences of the FMR line shift in an electric field were studied, showed that this microwave ME effect is much stronger for some other orientations of a bias field [59]. One of the possible explanations for this may be the need to take into account two-magnon scattering [60,62]. Another possible reason may be the accumulation of charges of the opposite sign on the surface of the magnetostrictive phase [63]. The final elucidation of the causes of this phenomenon requires additional research.

Above, following [67], we described the features of the microwave ME effect in the presence of a substrate, which are related to the need to take into account bending deformations and the clamping effect. But besides this, in the presence of a substrate, the microwave ME effect is also affected by the mismatch between the crystal lattices of the piezoelectric and the substrate [1]. Piezoelectric crystal lattices and substrates have different cell sizes. Using the phenomenological thermodynamic theory of Landau–Ginsburg–Devonshire, it was shown [88] that this leads to a change in the piezoelectric coefficients and the relative permittivity of the piezoelectric. A change in the piezoelectric coefficients naturally affects the magnitude of the microwave ME effect.

## 5. Conclusions

A general theory of the ME effect in composites in the low-frequency range, including the EMR region, is presented. The main EMR modes are considered in more detail: Longitudinal, bending, longitudinal-shear, and torsional. To demonstrate the theory, expressions are obtained for the ME voltage coefficients for symmetric and asymmetric layered structures. A comparison is made with the experimental results for the GaAs/Metglas and LiNbO_3_/Metglas structures. Thickness and thickness-shear modes of the ME effect, as well as wave piezoelectric phenomena, along with spin waves, we plan to consider in the future. For completeness of the analysis, a review of the main works on analytical methods for calculating the ME effect in the low-frequency range, including in the EMR region, is given.

On the basis of our previous works, we considered the general theory of the inverse ME effect in composites in the high-frequency range. The inverse ME effect is described as a composite FMR line shift in the external electric field. As an example, data on the calculation of the ME effect for the FM/piezoelectric and YIG/piezoelectric structures are given, where Ni is considered the FM and quartz, PZT, PMN-PT, and PZN-PT are considered piezoelectrics. The calculation of the “substrate effect” for the layered structure of the piezoelectric/YIG/GGG, which is important for experimental studies, is considered. In addition, the main works describing the analytical methods for calculating the inverse ME effect in the high-frequency region are given.

The works on the use of the FEM for calculating the ME effect in the low-frequency and high-frequency regions are analyzed. It is shown that for FEM modeling, this is still the initial stage, and the HFSS Ansoft and Comsol Multiphysics packages are most often used. The results obtained are in good agreement with the analytical methods and experiments.

## Data Availability

Not applicable.
